# Do psychosocial factors modify the negative association between disability and life satisfaction in old age?

**DOI:** 10.1371/journal.pone.0224421

**Published:** 2019-10-31

**Authors:** Thomas Puvill, Sasmita Kusumastuti, Rikke Lund, Erik Lykke Mortensen, Joris Slaets, Jolanda Lindenberg, Rudi G. J. Westendorp

**Affiliations:** 1 Leyden Academy on Vitality and Ageing, Leiden, the Netherlands; 2 Section of Epidemiology, Department of Public Health, University of Copenhagen, Copenhagen, Denmark; 3 Center for Healthy Aging, University of Copenhagen, Copenhagen, Denmark; 4 Section of Social Medicine, Department of Public Health, University of Copenhagen, Copenhagen, Denmark; 5 Section of Environmental Health, Department of Public Health, University of Copenhagen, Copenhagen, Denmark; 6 Department of Public Health and Primary Care, Leiden University Medical Center, Leiden, the Netherlands; Università degli Studi di Perugia, ITALY

## Abstract

**Context:**

Many assume that having poor physical health in old age lowers life satisfaction, but in fact there are large differences in life satisfaction among older people who experience disability.

**Objective:**

To investigate whether psychosocial factors modify the negative association between disability and life satisfaction in older people and whether these differ across the life course.

**Design:**

Cross sectional study.

**Setting:**

66,561 community-dwelling Survey of Health, Ageing, and Retirement in Europe (SHARE) participants aged 50–106 with a mean age of 67.8 ± 9.9 (SD) years from 17 European countries and Israel.

**Methods:**

Psychosocial factors included depression (EURO-D scale), perceived loneliness, having a spouse, having children, contact with children, and participation in social activities. Disability was assessed by limitations in (Instrumental) Activities of Daily Living ((I)ADL) and life satisfaction by Cantril’s ladder. We also ran the analyses with the Control Autonomy Self-realization Pleasure (CASP-12) Index, a normative measure of quality of life. We used multiple linear regressions to estimate associations and proportion of variance explained.

**Results:**

The variance in life satisfaction that could be attributed uniquely to ADL and IADL disability was 0.17% and 0.33% respectively (both p < 0.001). The impact of (I)ADL disabilities on life satisfaction was strongest at age 50 and gradually decreased with increasing age (p trend < 0.001). Mental health explained more variance; 5.75% for depressive symptoms and 2.50% for loneliness and for social resources this ranged from 0.09% to 0.47% (all p < 0.001). While disability has a negative effect on life satisfaction, the effect was not stronger in older persons who were depressed, neither in those who felt lonely nor in those without social resources. Similar outcomes were found when using CASP-12 as the explained variable.

**Conclusion:**

The impact of (I)ADL disabilities on life satisfaction in community-dwelling older people decreases with age. These associations are not affected by psychosocial factors and these patterns cannot be explained by people changing their norms and values.

## Introduction

People aspire to have a long and satisfactory life. Yet old age comes with infirmity, and many assume that living with disabilities lowers life satisfaction in old age. In fact, there are large differences in life satisfaction among older people facing disabilities,[1] and on a group-level the relationship between disabilities and life satisfaction is only small.[2] Psychosocial factors could play a role in these findings. [3–6] First, older people who suffer from mental health symptoms, such as depressive feelings and loneliness, seem especially susceptible to the detrimental effects of disabilities,[7] and this in turn may lower their life satisfaction. Second, people with many social resources cope better when struggling with health issues than those with few social resources,[8] and thus older people’s social resources may influence whether functional decline lowers life satisfaction.[9]

Although it is entirely plausible that psychosocial factors may modify the relationship between older people’s functional status and their life satisfaction, this has not yet been researched in community-dwelling older populations. [10,11] It is also not known whether such a modifying role of psychosocial factors might change over the lifespan.[12] Older age spans more than 30 years, and what holds true for the youngest old may not hold true for the oldest old. It is therefore possible that the interrelations among psychosocial factors, functional status and life satisfaction change over the life course. [13] An essential feature of life satisfaction is that this general evaluation of life is interpreted based on people’s own norms and values. These norms and values can change, for instance when people learn to accept and adapt to a new situation. [14]

The aim of the present study is to investigate whether psychosocial factors modify the association between disability and life satisfaction in older people and whether this modifying effect differs across the life course. Quality of life was included as a normative outcome to tease apart the modifying effect of psychosocial factors and a change in personal norms and values. To this end we use data from the SHARE study: a large-scale cohort study in 27 European countries and Israel.[15] The SHARE database provides a unique opportunity for our purpose, as its participants are within a large age range (50–106), and the exceptionally large sample size (> 66,000) allows us to investigate less prevalent subgroups that cannot be researched using other surveys. If psychosocial factors are found to modify the negative effect of disabilities on life satisfaction and quality of life, then additional interventions in these domains may be particularly helpful.

## Materials and methods

### Study population

This study was based on secondary data analysis of the Survey of Health, Ageing, and Retirement in Europe (SHARE); a longitudinal database on health, wellbeing, socio-economic status, and social networks of community-dwelling persons aged 50 years and older, as well as their partners independent of age living in the same household.[15] We constructed a study sample consisting of all participants who were interviewed in SHARE Wave 6 (2015) release version 6.0.0 (N = 68,231).[16,17] For the purpose of this study, spouses / partners younger than 50 (N = 897) and participants who lived in nursing homes (N = 773) were excluded.

### Procedure

Participants were excluded from SHARE survey if incarcerated, hospitalized, abroad during the survey period, or unable to speak the country’s language. Information on demographics, psychosocial factors, disabilities, life satisfaction, and quality of life was collected using a questionnaire that was translated into the country’s languages and processed automatically in a Computer-Assisted Personal Interviewing (CAPI) instrument. The interviewers conducted face-to-face interviews using a computer on which the CAPI instrument was installed.

### Demographic variables

Demographic variables included age, sex, country, educational levels, and household ability to make ends meet. Educational levels were categorized according to 1997 International Standard Classification of Education and further simplified to no education, basic education (primary/secondary), upper education (post-secondary), still in school / other.[18] Household ability to make ends meet was based on the answer to the question “Is the household able to make ends meet?” and categorized into having difficulty or no difficulty making ends meet. Although ethnic background may be relevant, unfortunately no information on ethnicity was available.

### Psychosocial factors

#### Mental health

Depression and experienced loneliness were included to investigate the modifying effects of mental health. Depression was measured with the EURO-D, [19] a 12-item questionnaire measuring prevalence of depression in older populations. The reliability of this measure ranged from Cronbach’s alpha 0.61 to 0.75.[20] Scores ranged from 0–12 and a score of > 3 points indicated depression. This variable was dichotomized into depressed and non-depressed. For experienced loneliness, SHARE used the Revised-University of California at Los Angeles Loneliness scale (R-UCLA), which had a reliability of 0.87.[21] The scale was based on the questions: “how often do you feel lack of companionship, feel left out, and feel isolated from others?” Here we divided experienced loneliness into not lonely and lonely.

#### Social resources

Social resources included having a spouse, having children, weekly contact with most contacted child, and participation in activities. Participants were asked whether they currently had a spouse and/or children. Contact with their children was estimated by asking participants for each of their children how often they and/or their partner had contact with a child in the past month. This contact could be face-to-face, by phone or e-mail. The child with the highest contact frequency was included in the analysis, and recoded into weekly or less than weekly. Participation in social activities was operationalized in the same way as in an earlier study for uniformity.[22] Participants were prompted to indicate on a response card whether they had participated in any of the following activities in the past month: a) voluntary or charity work, b) cared for a sick or disabled adult, c) provided help to friends or neighbours, d) attended an education or training course, e) gone to a sport, social or other kind of club, f) taken part in activities of a religious organization (church, synagogue, mosque, etc.). Here we categorized the variables into one or more and no activities.

### Disabilities

Participants were asked to indicate on a response card if they had any of the ten limitations in their Activities of Daily Living (ADL)[23], and on another response card if they had any of the fifteen limitations in their Instrumental Activities of Daily Living (IADL)[24] (see S1 and S2 Tables). Participants were asked to exclude any difficulties that they expected to last less than three months. Reported disabilities were added up so that possible scores ranged from 0 to 10 for ADL disabilities and 0 to 15 for IADL disabilities, and were treated as a continuous variable in the analyses. Both ADL and IADL had a reliability of 0.78.[25]

### Life satisfaction and quality of life

As outcome measures, we included life satisfaction and a quality of life measure. Life satisfaction was determined by the participant’s rating to a single item question “How satisfied are you with your life?” and was scaled continuously from 0 (completely dissatisfied) to 10 (completely satisfied) by the Cantril’s ladder.[26] Quality of life was measured using the 12-item Control Autonomy Self-realisation Pleasure (CASP-12) questionnaire,[27] a shorter version of the original CASP-19[28] (see S3 Table). For this measure, participants were asked about how often they experience particular feelings or situations on a 4-point scale ranging from ‘never’ to ‘often’, resulting in scores ranging from 12 to 48. The single item life satisfaction had a reliability of 0.6 as estimated using panel data and multivariate latent state-trait models[29] and the multiple items CASP-12 Index for Quality of Life had a reliability of 0.7 for Control, 0.3 for Autonomy, 0.9 for Self-realization, and 0.7 for Pleasure.[27] These latter two measures have a relatively strong Spearman correlation coefficient of 0.6.

### Data handling

Missing data largely ranged between 0% (age / sex / country / household income) to 6.3% (CASP Index for Quality of Life), except for one variable 20.1% (weekly contact with most contacted child) (see S4 Table). The latter percentage includes individuals who have never had children and drops to 17.3% if these individuals are left out of the sample. After taking this into account, the scatter plots showed that the item responses are missing at random, as were those for the other variables. Given the large sample more than enough power was left for the analyses. Since missing data was not higher than 5% for most of the variables we decided not to impute the data. From hereon we ran complete case analyses and missing items were left out of the analyses.

### Statistical analysis

First, main effects and their effect sizes (eta-squared) were calculated using univariate and multiple linear regressions. For the multiple linear regressions, estimates were adjusted for demographic variables, functional status, mental health variables, and social resources. We then investigated effect modifications by performing multiple linear regressions to examine the association between disability and life satisfaction stratified by the dichotomized psychosocial factors, adjusting for variables in the study. As another way to test effect modifications, we also utilized interaction terms. Dichotomization of the psychosocial variables was done as followed: depressed / not depressed, lonely / not lonely, having no spouse / a spouse, having no children / one or more children, less than weekly contact with child / weekly contact or more, no participation in activities / participation in one or more activities. To investigate the association between disability and life satisfaction across age groups, we divided age into categories of five years, resulting in nine age categories. Here we used two models: the simple model adjusted for demographics only (age, sex, and country), and the full model adjusted for demographics and psychosocial factors. In addition to analyses based on the age categories, trend analyses using the full age range of participants were used. In addition to life satisfaction, all analyses were performed a second time using quality of life as outcome. All statistical analyses were performed using R version 3.4.0.[30]

## Results

### Characteristics of the study sample

Table 1 presents characteristics of the study sample. In total, we included 66,561 community-dwelling participants from 18 countries: northern- (Denmark, Sweden), southern- (Portugal, Spain, Italy, Greece), western- (Belgium, Luxembourg, France, Germany, Austria, Switzerland), eastern Europe (Czech Republic, Poland, Estonia, Slovenia, Croatia), and Israel. Participants’ age ranged from 50–106 years old with average age of 67.8 ± 9.9 (SD) years. Of the total study sample, 55.9% were females, 67.0% attended basic primary or secondary education, and 38.2% had difficulty making ends meet. ADL and IADL disabilities occurred in respectively 11.7% and 18.3% of the study sample. As markers of mental health, participants had a median of two depressive symptoms out of a scale of 12 and 44.0% experienced loneliness. In terms of social resources, 71.3% of the study sample had a spouse and the median for having children was two. Around 65.7% had weekly contact with their child and the median number of activities participated in was two. Participants reported high levels of life satisfaction with a median of eight out of ten and quality of life with a median score of 38 out of 48.

**Table 1:** Characteristics of the study sample.

Characteristics	
Total number of participants	66,561 [100·0]
Demographic Variables	
Age, mean (SD)	67·8 (9·9)
Female	37,224 [55·9]
Basic education	43,989 [67·0]
Having difficulties making ends meet	25,398 [38·2]
Disabilities	
Has disabilities in ADL	7,775 [11·7]
Has disabilities in IADL	12,124 [18·3]
Psychosocial Factors	
Mental Health	
Depressive symptoms, median (IQR)	2 (3)
Experiencing loneliness	27,987 [44·0]
Social Resources	
Having a spouse	47,025 [71·3]
Number of children, median (IQR)	2 (2)
Weekly contact with child	43,709 [65·7]
Number of activities participated, median (IQR)	2 (2)
Life satisfaction in 10-point scale, median (IQR)	8 (2)
CASP Index for Quality of Life, median (IQR)	38 (9)

Data are presented as number of participants [percentage] except as noted.

### The main effects

Table 2 shows the main effects of functional status, psychosocial factors, and demographics on life satisfaction and quality of life. Most of these factors were associated at p < 0.001 and effect sizes were overall larger for quality of life compared to life satisfaction. Each increase in ADL-points decreased life satisfaction with -0.12 points and quality of life with -0.38 point, and for IADL the decreases were -0.10 points and -0.69 points respectively. Mental health has the biggest negative effect: depressive symptoms lowered life satisfaction with -0.93 points and quality of life with -3.83 points followed by experienced loneliness with -0.54 and -2.69 points respectively. Regarding proportion of variance explained, variance in life satisfaction and quality of life that can be attributed uniquely to ADL was 0.17% and 0.20%, while for IADL disability it was 0.33% and 1.58% respectively. Mental health explained more of the variance in life satisfaction and in quality of life: for depressive symptoms, this was 5.75% and 10.41%, and for experienced loneliness this was 2.50% and 6.69% respectively. Explained variance for social resources was smaller, ranging from 0.00% to 1.47%. The explained variance of mental health and social resources were probably somewhat underestimated because these predictor variables were dichotomous. Of the demographic variables, country explained most variance, 3.54% for life satisfaction and 7.59% for quality of life followed by household ability to make ends meet which contributed 2.85% for life satisfaction and 6.25% for quality of life. In an exploratory analysis (see S5 Table) effect sizes for explained variances were notably larger for mental health and functional status when we look at the crude, unadjusted analyses only.

**Table 2:** The effects of functional status, psychosocial factors, and demographics on life satisfaction and quality of life.

Explanatory Variables	Life Satisfaction Range 0–10		CASP-12 Index for Quality of Life Range 12–48	
	Change [SE]	Proportion of Variance Explained	Change [SE]	Proportion of Variance Explained
**Functional status**				
Per ADL disability	-0.12 [0.01] ***	0.17%	-0.38 [0.03] ***	0.20%
Per IADL disability	-0.10 [0.01] ***	0.33%	-0.69 [0.02] ***	1.58%
**Psychosocial factors**				
Mental health				
Depressed^a^	-0.93 [0.02] ***	5.75%	-3.83 [0.05] ***	10.41%
Experienced loneliness^a^	-0.54 [0.01] ***	2.50%	-2.69 [0.04] ***	6.69%
Social resources				
No spouse^a^	-0.26 [0.02] ***	0.47%	0.06 [0.05]	0.00%
No children^a^	-0.19 [0.03] ***	0.09%	-0.42 [0.08] ***	0.05%
Less than weekly contact with child^a^	-0.20 [0.02] ***	0.14%	-0.32 [0.07] ***	0.05%
No participation in activities^a^	-0.26 [0.02] ***	0.32%	-1.66 [0.06] ***	1.47%
**Demographics**				
Age per 5 years increase	0.05 [0.00] ***	0.41%	-0.20 [0.01] ***	0.61%
Sex: Female	0.17 [0.01] ***	0.28%	0.22 [0.04] ***	0.05%
Country	na	3.55%	na	7.59%
Educational levels: basic education	0.05 [0.03]	0.03%	0.52 [0.09] ***	0.29%
Household ability to make ends meet: difficult	-0.37 [0.05] ***	2.85%	-1.90 [0.15] ***	6.25%

Data presented as regression coefficients [standard error] and partial eta-squared from multiple linear regressions adjusted for demographics, functional status, and psychosocial factors.

na: no estimates for individual countries.

^a^ Variables were dichotomized into yes and no responses.

Significance * *p* < 0.05, ** *p* < 0.01, *** *p* < 0.001

### The modifying effects

The strength of the relationship between (I)ADL disability and life satisfaction and quality of life remained largely unmodified by differences in psychosocial factors, except for an extremely small, but significant effect of depression and experienced loneliness. In addition to marital status, we looked at the modifying effects of cohabitation status for exploratory purposes. We found that living alone or with others also did not change the association between (I)ADL disabilities and life satisfaction, and results were the same when using quality of life as the outcome variable. All associations between (I)ADL disability, life satisfaction and quality of life and the modifying effects of psychosocial factors and demographics are shown in S6 and S7 Tables. We also performed the same analyses separate for males and females and results were similar. Formal testing for interaction of the latter in general did not reveal significant results, see S8 Table.

**Fig 1:**
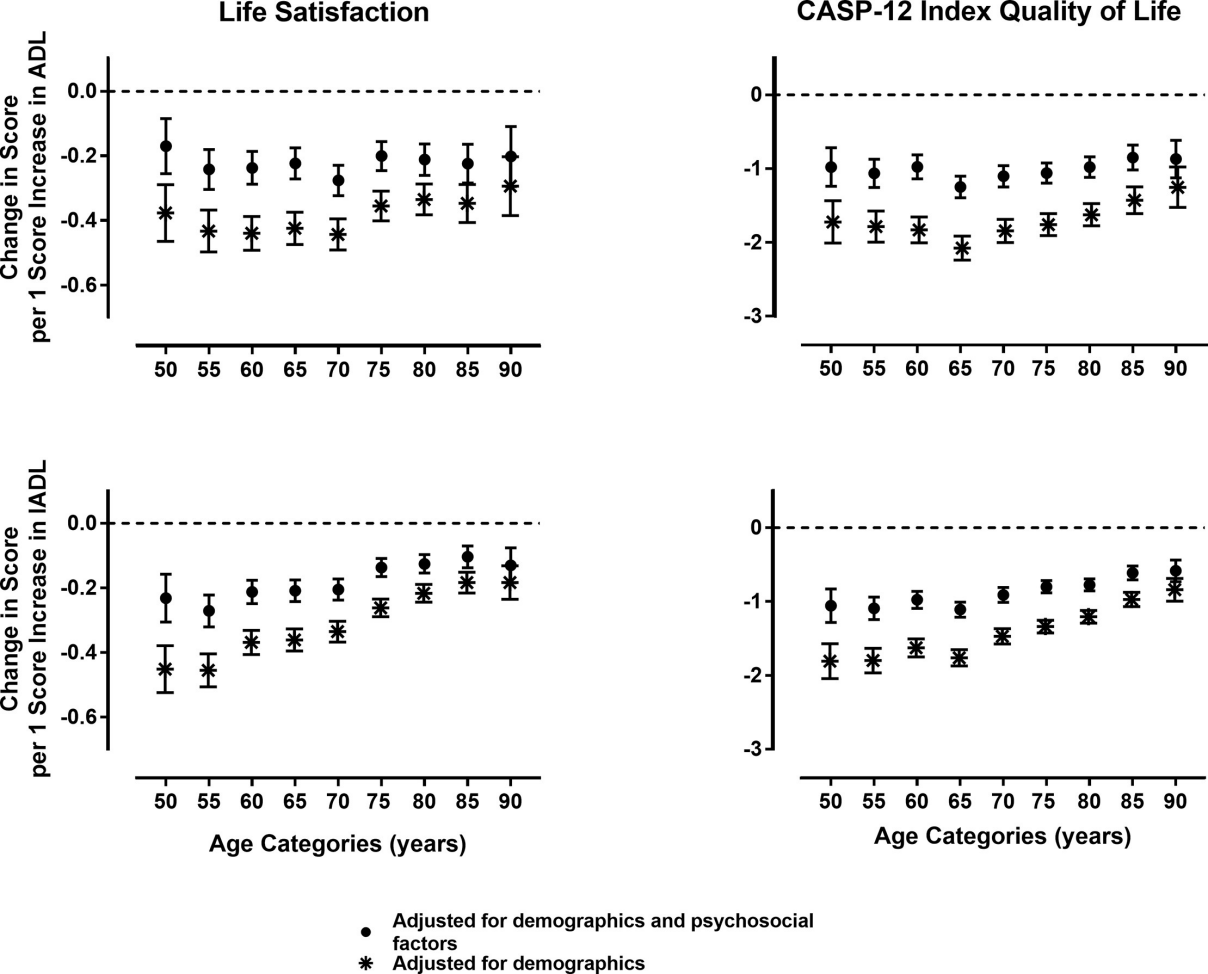
The Association between (I)adl disability, life satisfaction and quality of life over age categories. Data presented are from regression coefficients and 95% confidence intervals from separate multiple linear regressions.

Fig 1 shows the effect of (I)ADL disabilities on life satisfaction and quality of life over age categories using regression coefficients and 95% confidence intervals. Here we show two models: the simple model adjusted for demographics only, while the full model is adjusted for demographics and psychosocial factors. In general, increase in (I)ADL disability leads to lower levels of life satisfaction and quality of life but this association is smaller in the full model. The impact of I(ADL) disabilities on life satisfaction was strongest at age 50 and gradually decreased with increasing age. Similar trends are observed when using quality of life as the outcome, with lowest effect sizes in the oldest age categories. Trend analyses were overall significant especially for IADL over age with *P* trend < 0.001 for all models, as for ADL the simpler models revealed significant *P* trend < 0.001.

We formally tested for three-way interaction between disability, life satisfaction and quality of life, and the modifying effects of psychosocial factors over age categories, and although some of the p-values were significant, the effect sizes were extremely small, see S9 Table. This indicates that the effect of mental health and social resources on the association between disabilities, life satisfaction and quality of life remained largely unaltered over age.

## Discussion

This study investigates whether psychosocial factors modify the negative association between disability, life satisfaction and quality of life in community-dwelling older people living in European countries and Israel, and whether this effect differs over the life course. First, mental health factors and social resources are all significantly associated with life satisfaction and quality of life, but only mental health factors have a substantial effect. Second, none of these psychosocial factors modified the association between (I)ADL disabilities, life satisfaction and quality of life. This lack of influence is observed across all age categories. Third, we find that the associations between disabilities, life satisfaction and quality of life decrease with age, suggesting that disabilities lose their negative impact to enjoy a good life in later life.

### Normative versus subjective

The pattern of results, especially the effect modification by social factors, is the same whether we use a global measure of life satisfaction, which due to its unspecificity relies on criteria chosen and weighed by the respondent, or the CASP-12 Index which is a normative measure of quality of life. These similar patterns suggest that the lack of modification by psychosocial factors on the relation between disability and life satisfaction is more than just older individuals changing their norms and values. [14]

The one discrepancy we observed between life satisfaction and quality of life was that the effect sizes for some of the disabilities, psychosocial variables and demographic variables are larger for quality of life than for life satisfaction. This could be due to conceptual differences between quality of life and life satisfaction, or a consequence of the different approach of an individually weighted versus a standardized rating; the latter does not allow for changes in norms and values that in real life occur when people adapt to their situation or get used to it. This adaptation resonates in numerous theories postulating that people are satisfied when their perceived reality aligns with their desire or expectations[31–35], and that people can adjust to a gap between reality and expectations by shifting their norms downwards[34,36], or by adopting new values altogether[37,38]. We therefore postulate that the effect sizes between disabilities and quality of life are stronger, as this standardized scale included items such as “I feel full of energy these days” which are closer to functional ability than life satisfaction as appreciated by an individual. Another possible explanation is the difference in reliability between life satisfaction and quality of life.[27,29] Life satisfaction is a single-item score and therefore tends to have lower reliability (0.6)[29] compared to multiple-items such as in the CASP-12 with reliability of 0.7 for Control, 0.3 for Autonomy, 0.9 for Self-realization, and 0.7 for Pleasure.[27] These differences may explain why there are stronger associations for quality of life.

### Main associations

The overall negative associations between disability and life satisfaction that are reported here have been reported earlier in other studies among community-dwelling adults.[39–41] Our study confirms these relationships and on top of this provides effect sizes showing that although the main effects of disability, psychosocial factors, and demographics on life satisfaction were significant, disabilities and social resources explain only little variance in older people’s life satisfaction, with mental health factors explaining most variance.

Finally, although outside of the scope of the current paper, a notable finding is that out of the demographic variables, country of residence explained a relatively large amount of the unique variance in our models, as well as household income in the supplementary univariate analysis. Speculating, this pattern reminds of Diener et al.’s proposition that wealthier countries report a higher life satisfaction and quality of life than less wealthy countries, because a population with a higher standard of living is better able to fulfil its basic needs. This, they argue, enables inhabitants of wealthier countries to focus on improving other domains of life, thus leading to higher satisfaction and quality of life.[42] Other explanations for the large between-country differences could be differences in cultural norms and values. An alternative explanation lies in the fact that the psychosocial variables depression and experience of loneliness, and possibly disabilities in (I)ADL, are subjective measures, as are the outcome measures quality of life and life satisfaction. Because of this, other factors such as the participants’ general mood at the time of responding may inflate the correlation between the predictor and outcome variables. This could also explain why the more objective measures such as marital status and having children have a smaller correlation. One study that modelled the variance of one-item life satisfaction questions from several large longitudinal studies,[43] showed that 5% and 13% of variance could be attributed to participants’ state during that wave, which is likely to include both ‘actual’ fluctuations in life satisfaction, and factors specific to the moment of measurement such as mood state. Although we do not know if third factors inflate the relationship between the subjective predictors and life satisfaction and quality of life, we do have indication that such situational third factors only have a limited association with these outcome measures, and therefore we carefully speculate that confounding by third factors as mood should be acceptable.

### Effect modification

Contrary to expectations, the negative association between disabilities, life satisfaction and quality of life was largely unmodified by the studied psychosocial variables. This essential finding seems to be discordant with theories on social support which state that people with many social resources cope better when struggling with health issues than those with few.[8] However, older people may care more about quality than quantity[44], and the lack of effect modification may reflect that we did not have the most valued social resource variables at our disposal (see strengths and limitations). For mental health, our findings are discordant with the proposition that a good mental health is a resource that can be used to overcome the challenges posed by poor health[45]. This could be because, as we showed earlier[46], disabilities may be the only aspect of physical health that cannot be compensated for by mental health in maintaining life satisfaction, precisely because of the restrictive nature of disabilities on other aspects of life.

### Age effects

The association between disabilities and life satisfaction that we report here is only modest, with the smallest association found in the oldest age categories. Our earlier findings are in accordance with this: in a different group of older people, we found that physical health is relatively unimportant for life satisfaction at advanced age[46]. However, to our knowledge, the age-related decline in this already weak association has not been shown before. These results are not entirely surprising, as it has been shown repeatedly that longitudinal trajectories of life satisfaction do not run parallel to trajectories of physical decline at old age[47] and that physical health becomes increasingly unimportant to how older people evaluate their health[48]. This age-specific effect can occur when older people compare themselves with age-peers who are equally or more disabled[33,34,38]; thus adopting a more accepting attitude towards their own disabilities.

### Effect modification in simple versus full model

Association between disabilities, life satisfaction and quality of life become even smaller when adjusting for personal characteristics compared to the simpler models. This is the same for all age groups, although the models seem to converge at old age. As mental health variables were the only psychosocial variables with a substantial association with life satisfaction and quality of life, we infer that depressive symptoms and loneliness account for most of the difference between the simple and full model. In other words, a large share of the covariance between disabilities, life satisfaction and quality of life cannot be uniquely attributed to disabilities, but is in fact shared covariance with mental health, as was already shown earlier using data from the Leiden 85-plus Study.[2] This lower shared, but not unique variance at old age, could indicate that the true driver between the decreasing association between disabilities, life satisfaction and quality of life is that in old age, depressive symptoms occur more often in those without disabilities, or disabilities less frequently co-occur with depressive symptoms.

### Strengths and limitations

A major strength of this study is that we used a very large sample size, allowing us to use age-stratification and sub-group analysis with sufficient precision. We increased the generalizability of our findings to developed countries by including many European countries. An important limitation of current study is that all analyses were cross-sectional. Because of this, we are unable to rule out alternative explanations for our findings. For instance, we cannot reject the possibility that the decreasing importance of functional status to life satisfaction at older age was a cohort effect rather than an age-specific effect. Another limitation is that the information on social resources does not necessarily cover what older people find important. For instance, older people find quality of relationships more important than quantity,[5] and therefore the amount of participation in social activities may not necessarily be meaningful to older individuals. Moreover, participation in social activities is low in all age categories, suggesting that the included social activities may not be important to older people at all, let alone missed in the face of functional decline. Having a spouse or offspring also does not necessarily cover what older people find important, as many may still experience high quality social contacts from other social resources, whereas marital and parental relationships may also be experienced as a source of stress. The mental health variables included have the limitation that their covariance with life satisfaction could be confounded by third factors such as mood or personality. This warrants caution in interpreting the effect size of the relationship between mental health and life satisfaction, for which future studies could use the longitudinal data of SHARE. We also note that the loneliness question was quite direct and may have yielded socially desirable answers. Another limitation would be that there are self-reported measures which are much more dependent on the general mood of the respondent (e.g. life satisfaction, quality of life, depression, experience of loneliness) and those that are less dependent (e.g. having a spouse or not, having children or not). In this case, depression is expected to show stronger associations to the outcome. However, in our study these associations are relatively weak and this may be because the EURO-D and the quality of life measures contain overlapping items (how the person looks at the future, having too little energy, etc.).

### Implications

This research showed that mental health had a far stronger association with life satisfaction than disabilities and social resources, indicating that focusing on mental health interventions to improve life satisfaction may be particularly fruitful. However, it would be wrong to conclude that social resources and functional status are unimportant to older people: older people consider social resources and functional health very important in their life.[49,50] These apparently contradictory findings from the present study indicate that older people with disabilities or few social resources can adapt to these circumstances when they are faced with disabilities and or lack social resources.

## Supporting information

S1 Table Variables for constructing limitations in activities of daily living. (DOCX)

S2 Table Variables for Constructing Limitations in Instrumental Activities of Daily Living. (DOCX)

S3 Table Variables for Constructing SHARE Version of CASP-12 Index for Quality of Life. (DOCX)

S4 Table Percentage of Missing Data. * This percentage includes people without children.
(DOCX)

S5 Table The Effects of Functional Status, Psychosocial Factors, and Demographics on Life Satisfaction and Quality of Life. Data presented as regression coefficients [standard error] and eta-squared from univariate linear regressions. na: no estimates for individual countries. ^a^ Variables were dichotomized into yes and no responses. Significance * *p* < 0.05, ** *p* < 0.01, *** *p* < 0.001.
(DOCX)

S6 Table The Association between (I)ADL Disability, Life Satisfaction and Quality of Life Stratified by Psychosocial Factors and Demographics. Data presented are from separate multiple linear regressions adjusted for demographics, functional status, and psychosocial factors. Formal testing for interaction revealed some significant results, however the effect sizes were very small (see S7 Table).
(DOCX)

S7 Table Two-way Interaction Analysis of Disability–Psychosocial Factors on Life Satisfaction and Quality of Life. Significance * p < 0.05, ** p < 0.01, *** p < 0.00 Data presented are adjusted for demographics and other psychosocial factors. Full range of the variables were used.
(DOCX)

S8 Table Three-way Interaction Analysis of Disability–Psychosocial Factors–Sex on Life Satisfaction and Quality of Life. Significance * *p* < 0.05, ** *p* < 0.01, *** *p* < 0.00 Data presented are adjusted for demographics and other psychosocial factors. Full range of the variables were used.
(DOCX)

S9 Table Three-way Interaction Analysis of Disability–Psychosocial Factors–Age on Life Satisfaction and Quality of Life. Significance * *p* < 0.05, ** *p* < 0.01, *** *p* < 0.00 Data presented are adjusted for demographics and other psychosocial factors. Full range of the variables were used.
(DOCX)
