# Supplementary material for: Do psychosocial factors modify the negative association between disability and life satisfaction in old age?
Source: PLoS One. 2019 Oct 31;14(10):e0224421. doi: 10.1371/journal.pone.0224421 (PMC6822713; doi:10.1371/journal.pone.0224421)
Supplement: S1 Table — (DOCX) [file pone.0224421.s001.docx]

**S1 Table. Variables for Constructing Limitations in Activities of Daily Living**

| **Items** | **Variable** |
| --- | --- |
| 1 | Walking 100 metres |
| 2 | Sitting for about two hours |
| 3 | Getting up from a chair after sitting for long periods |
| 4 | Climbing several flights of stairs without resting |
| 5 | Climbing one flight of stairs without resting |
| 6 | Stooping, kneeling, or crouching |
| 7 | Reaching or extending your arms above shoulder level |
| 8 | Pulling or pushing large objects like a living room chair |
| 9 | Lifting or carrying weights over 10 pounds / 5 kilos, like a heavy bag of groceries |
| 10 | Picking up a small coin from a table |
