# Supplementary material for: Do psychosocial factors modify the negative association between disability and life satisfaction in old age?
Source: PLoS One. 2019 Oct 31;14(10):e0224421. doi: 10.1371/journal.pone.0224421 (PMC6822713; doi:10.1371/journal.pone.0224421)
Supplement: S2 Table — (DOCX) [file pone.0224421.s002.docx]

**S2 Table. Variables for Constructing Limitations in Instrumental Activities of Daily Living**

| **Items** | **Variable** |
| --- | --- |
| 1 | Dressing, including putting on shoes and socks |
| 2 | Walking across a room |
| 3 | Bathing or showering |
| 4 | Eating, such as cutting up your food |
| 5 | Getting in or out of bed |
| 6 | Using the toilet, including getting up or down |
| 7 | Using a map to figure out how to get around in a strange place |
| 8 | Preparing a hot meal |
| 9 | Shopping for groceries |
| 10 | Making telephone calls |
| 11 | Taking medications |
| 12 | Doing work around the house or garden |
| 13  14  15 | Managing money, such as paying bills and keeping track of expenses  Leaving the house independently and accessing transportation services  Doing personal laundry |
