# Supplementary material for: Do psychosocial factors modify the negative association between disability and life satisfaction in old age?
Source: PLoS One. 2019 Oct 31;14(10):e0224421. doi: 10.1371/journal.pone.0224421 (PMC6822713; doi:10.1371/journal.pone.0224421)
Supplement: S3 Table — (DOCX) [file pone.0224421.s003.docx]

**S3 Table. Variables for Constructing SHARE Version of CASP-12 Index for Quality of Life**

| **Dimensions** | **Items Variable** | |
| --- | --- | --- |
| Control | 1 | My age prevents me from doing the things I would like to do |
|  | 2 | I feel that what happens to me is out of my control |
|  | 3 | I feel left out of things |
| Autonomy | 4 | I can do the things I want to do |
|  | 5 | Family responsibilities prevent me from doing the things I want to do |
|  | 6 | Shortage of money stops me from doing things I want to do |
| Self - realization | 7 | I feel full of energy these days |
|  | 8 | I feel that life is full of opportunities |
|  | 9 | I feel that the future looks good for me |
| Pleasure | 10 | I look forward to each day |
|  | 11 | I feel that my life has meaning |
|  | 12 | On balance, I look back on my life with a sense of happiness |
