# Supplementary material for: Do psychosocial factors modify the negative association between disability and life satisfaction in old age?
Source: PLoS One. 2019 Oct 31;14(10):e0224421. doi: 10.1371/journal.pone.0224421 (PMC6822713; doi:10.1371/journal.pone.0224421)
Supplement: S4 Table — * This percentage includes people without children. (DOCX) [file pone.0224421.s004.docx]

**S4 Table. Percentage of Missing Data**

| **Variable** | **Percentage of Missing Data** |
| --- | --- |
| Age | 0 % |
| Sex | 0 % |
| Country | 0 % |
| Education | 1.3 % |
| Household ability to make ends meet | 0 % |
| ADL Disability | 0.2 % |
| IADL Disability | 0.2 % |
| Depressive symptoms | 4.8 % |
| Experiencing loneliness | 4.4 % |
| Having a spouse | 0.9 % |
| Number of children | 0.4 % |
| Weekly contact with child | 20.1 % * |
| Number of activities participated | 4.0 % |
| Life satisfaction | 4.3 % |
| CASP Index for Quality of Life | 6.3 % |

* This percentage includes people without children
