# Supplementary material for: Do psychosocial factors modify the negative association between disability and life satisfaction in old age?
Source: PLoS One. 2019 Oct 31;14(10):e0224421. doi: 10.1371/journal.pone.0224421 (PMC6822713; doi:10.1371/journal.pone.0224421)
Supplement: S5 Table — Data presented as regression coefficients [standard error] and eta-squared from univariate linear regressions. na: no estimates for individual countries. a Variables were dichotomized into yes and no responses. Significance * p < 0.05, ** p < 0.01, *** p < 0.001. (DOCX) [file pone.0224421.s005.docx]

**S5 Table. The Effects of Functional Status, Psychosocial Factors, and Demographics on Life Satisfaction and Quality of Life**

| **Explanatory Variables** | **Life Satisfaction**  **Range 0 - 10** | |  | **CASP-12 Index for Quality of Life**  **Range 12 - 48** | |
| --- | --- | --- | --- | --- | --- |
|  | **Change**  **[SE]** | **Proportion of Variance Explained** |  | **Change**  **[SE]** | **Proportion of Variance Explained** |
| **Functional status** |  |  |  |  |  |
| Per ADL disability | -0.47 [0.01] *** | 3.76 % |  | -2.32 [0.03] *** | 6.96 % |
| Per IADL disability | -0.31 [0.01] *** | 4.62 % |  | -1.75 [0.02] *** | 11.03 % |
| **Psychosocial factors** |  |  |  |  |  |
| Mental health |  |  |  |  |  |
| Depressed^a^ | -1.43 [0.01] *** | 12.84 % |  | -6.39 [0.05] *** | 20.16 % |
| Experienced loneliness^a^ | -1.06 [0.01] *** | 8.79 % |  | -5.31 [0.05] *** | 17.41 % |
| Social resources |  |  |  |  |  |
| No spouse^a^ | -0.56 [0.02] *** | 2.03 % |  | -1.54 [0.06] *** | 1.20 % |
| No children^a^ | -0.29 [0.02] *** | 0.23 % |  | -0.58 [0.09] *** | 0.07 % |
| Less than weekly contact with child^a^ | -0.34 [0.03] *** | 0.37 % |  | -0.62 [0.09] *** | 0.10 % |
| No participation in activities^a^ | -0.81 [0.02] *** | 2.89 % |  | -5.09 [0.06] *** | 9.15 % |
| **Demographics** |  |  |  |  |  |
| Age per 5 years increase | -0.02 [0.00] *** | 0.03 % |  | -0.52 [0.01] *** | 2.58 % |
| Sex: Female | - 0.13 [0.01] *** | 0.12 % |  | -0.84 [0.05] *** | 0.43 % |
| Country | na | 8.52 % |  | na | 16.67 % |
| Educational levels: basic education | 0.60 [0.03] *** | 1.58 % |  | 4.34 [0.11] *** | 5.63 % |
| Household ability to make ends meet: difficult | -0.36 [0.05] *** | 10.19 % |  | -1.98 [0.18] *** | 17.43 % |

Data presented as regression coefficients [standard error] and eta-squared from univariate linear regressions.

na: no estimates for individual countries.

^a^ Variables were dichotomized into yes and no responses.

Significance * *p* < 0.05, ** *p* < 0.01, *** *p* < 0.001
