# Supplementary material for: Do psychosocial factors modify the negative association between disability and life satisfaction in old age?
Source: PLoS One. 2019 Oct 31;14(10):e0224421. doi: 10.1371/journal.pone.0224421 (PMC6822713; doi:10.1371/journal.pone.0224421)
Supplement: S6 Table — Data presented are from separate multiple linear regressions adjusted for demographics, functional status, and psychosocial factors. Formal testing for interaction revealed some significant results, however the effect sizes were very small (see S7 Table). (DOCX) [file pone.0224421.s006.docx]

**S6 Table. The Association between (I)ADL Disability, Life Satisfaction and Quality of Life Stratified by Psychosocial Factors and Demographics**

| **Explanatory Variables** | **N** | **Life Satisfaction**  **Range 0 - 10** | | | |  | **CASP-12 Index for Quality of Life**  **Range 12 – 48** | | | | |
| --- | --- | --- | --- | --- | --- | --- | --- | --- | --- | --- | --- |
|  |  | **ADL** | | **IADL** | |  | **ADL** | | **IADL** | | |
|  |  | **Coefficient (95% CI)** | **P-value** | **Coefficient (95% CI)** | **P-value** |  | **Coefficient (95% CI)** | **P-value** | **Coefficient (95% CI)** | **P-value** | |
| **Psychosocial Factors** |  |  |  |  |  |  |  |  |  |  | |
| Depression |  |  |  |  |  |  |  |  |  |  | |
| Depressed | 17,280 | -0.12 (-0.15 - -0.08) | < 0.001 | -0.12 (-0.14 - -0.10) | < 0.001 |  | -0.31 (-0.40 - -0.21) | < 0.001 | -0.69 (-0.75 - -0.63) | < 0.001 |  |
| Not depressed | 46,089 | -0.10 (-0.14 - -0.07) | < 0.001 | -0.07 (-0.09 - -0.05) | < 0.001 |  | -0.50 (-0.61 - -0.40) | < 0.001 | -0.67 (-0.74 - -0.61) | < 0.001 |  |
| Experienced loneliness |  |  |  |  |  |  |  |  |  |  | |
| Experiences loneliness | 35,657 | -0.14 (-0.17 - -0.11) | < 0.001 | -0.11 (-0.13 - -0.09) | < 0.001 |  | -0.40 (-0.48 - -0.31) | < 0.001 | -0.63 (-0.68 - -0.60) | < 0.001 |  |
| Not experiencing loneliness | 27,987 | -0.07 (-0.11 - -0.04) | < 0.001 | -0.10 (-0.12 - -0.07) | < 0.001 |  | -0.37 (-0.49 - -0.26) | < 0.001 | -0.80 (-0.87 - -0.73) | < 0.001 |  |
| Spouse |  |  |  |  |  |  |  |  |  |  | |
| No spouse | 18,908 | -0.14 (-0.18 - -0.11) | < 0.001 | -0.08 (-0.11 - -0.06) | < 0.001 |  | -0.34 (-0.45 - -0.24) | < 0.001 | -0.65 (-0.72 - -0.59) | < 0.001 |  |
| Have a spouse | 47,025 | -0.10 (-0.12 - -0.07) | < 0.001 | -0.12 (-0.14 - -0.11) | < 0.001 |  | -0.40 (-0.49 - -0.31) | < 0.001 | -0.71 (-0.77 - -0.65) | < 0.001 |  |
| Children |  |  |  |  |  |  |  |  |  |  | |
| No child | 6,491 | -0.07 (-0.14 -- 0.00) | 0.1 | -0.08 (-0.13 - -0.04) | < 0.01 |  | -0.36 (-0.57 -- 0. 14) | 0.001 | -0.59 (-0.72 - -0.45) | < 0.001 |  |
| Have one or more children | 59,827 | -0.13 (-0.15 - -0.10) | < 0.001 | -0.11 (-0.12 - -0.09) | < 0.001 |  | -0.39 (-0.46 - -0.31) | < 0.001 | -0.70 (-0.74 - -0.65) | < 0.001 |  |
| Contact with child |  |  |  |  |  |  |  |  |  |  | |
| Less than weekly | 5,774 | -0.11 (-0.18 - -0.04) | 0.001 | -0.10 (-0.14 - -0.05) | < 0.001 |  | -0.30 (-0.51 - -0.09) | 0.001 | -0.71 (-0.85 - -0.58) | < 0.001 |  |
| Weekly | 43,709 | -0.13 (-0.16 - -0.10) | < 0.001 | -0.11 (-0.12 - -0.05) | < 0.001 |  | -0.39 (-0.48 - -0.31) | < 0.001 | -0.70 (-0.76 - -0.65) | < 0.001 |  |
| Participation in activities |  |  |  |  |  |  |  |  |  |  | |
| No participation in activities | 10,983 | -0.16 (-0.21 - -0.11) | < 0.001 | -0.07 (-0.10 - -0.04) | < 0.001 |  | -0.36 (-0.50 - -0.23) | < 0.001 | -0.54 (-0.62 - -0.45) | < 0.001 |  |
| Participate in activities | 52,894 | -0.10 (-0.13 - -0.08) | < 0.001 | -0.12 (-0.13 - -0.10) | < 0.001 |  | -0.40 (-0.48 - -0.32) | < 0.001 | -0.75 (-0.81 - -0.70) | < 0.001 |  |
|  |  |  |  |  |  |  |  |  |  |  | |
| **Demographics** |  |  |  |  |  |  |  |  |  |  | |
| Educational levels |  |  |  |  |  |  |  |  |  |  | |
| Basic education | 43,989 | -0.12 (-0.15 - -0.10) | < 0.001 | -0.10 (-0.12 - -0.08) | < 0.001 |  | -0.39 (-0.47 - -0.30) | < 0.001 | -0.71 (-0.76 - -0.66) | < 0.001 |  |
| Upper education | 17,271 | -0.06 (-0.12 - -0.01) | 0.01 | -0.14 (-0.17 - -0.11) | < 0.001 |  | -0.40 (-0.57 - -0.23) | < 0.001 | -0.72 (-0.83 - -0.62) | < 0.001 |  |
| Household ability to make ends meet |  |  |  |  |  |  |  |  |  |  | |
| Having difficulty | 25,398 | -0.15 (-0.18 - -0.11) | < 0.001 | -0.10 (-0.11 - -0.07) | < 0.001 |  | -0.31 (-0.40 - -0.21) | < 0.001 | -0.65 (-0.71 - -0.58) | < 0.001 |  |
| No difficulty | 39,757 | -0.08 (-0.11 - -0.05) | < 0.001 | -0.12 (-0.14 - -0.10) | < 0.001 |  | -0.48 (-0.57 - -0.38) | < 0.001 | -0.72 (-0.78 - -0.65) | < 0.001 |  |

Data presented are from separate multiple linear regressions adjusted for demographics, functional status, and psychosocial factors.

Formal testing for interaction revealed some significant results, however the effect sizes were very small (see S7 Table)
