# Supplementary material for: Do psychosocial factors modify the negative association between disability and life satisfaction in old age?
Source: PLoS One. 2019 Oct 31;14(10):e0224421. doi: 10.1371/journal.pone.0224421 (PMC6822713; doi:10.1371/journal.pone.0224421)
Supplement: S7 Table — Significance * p < 0.05, ** p < 0.01, *** p < 0.00 Data presented are adjusted for demographics and other psychosocial factors. Full range of the variables were used. (DOCX) [file pone.0224421.s007.docx]

**S7 Table. Two-way Interaction Analysis of Disability – Psychosocial Factors on Life Satisfaction and Quality of Life**

|  | **ADL** | **IADL** |
| --- | --- | --- |
| **Life Satisfaction** |  |  |
| Depression | -0.021 *** | -0.013 *** |
| Experienced loneliness | -0.016 ** | -0.002 |
| Having a spouse | 0.013 * | 0.009 ** |
| Having children | -0.009 | 0.002 |
| Weekly contact with child | 0.006 | 0.003 |
| Participation in activities | 0.017 * | -0.002 |
|  |  |  |
| **CASP-12 Index for Quality of Life** |  |  |
| Depression | 0.050 *** | 0.032 *** |
| Experienced loneliness | 0.126 *** | 0.107 *** |
| Having a spouse | 0.028 * | 0.027 ** |
| Having children | -0.005 | 0.013 |
| Weekly contact with child | 0.020 | 0.011 |
| Participation in activities | 0.002 | -0.013 |

Significance * *p* < 0.05, ** *p* < 0.01, *** *p* < 0.00

Data presented are adjusted for demographics and other psychosocial factors.

Full range of the variables were used.
