# Supplementary material for: Do psychosocial factors modify the negative association between disability and life satisfaction in old age?
Source: PLoS One. 2019 Oct 31;14(10):e0224421. doi: 10.1371/journal.pone.0224421 (PMC6822713; doi:10.1371/journal.pone.0224421)
Supplement: S8 Table — Significance * p < 0.05, ** p < 0.01, *** p < 0.00 Data presented are adjusted for demographics and other psychosocial factors. Full range of the variables were used. (DOCX) [file pone.0224421.s008.docx]

**S8 Table. Three-way Interaction Analysis of Disability – Psychosocial Factors – Sex on Life Satisfaction and Quality of Life**

|  | **ADL** | **IADL** |
| --- | --- | --- |
| **Life Satisfaction** |  |  |
| Depression | 0.005 | 0.007 |
| Experienced loneliness | -0.015 | 0.004 |
| Having a spouse | 0.004 | 0.018 * |
| Having children | 0.018 | 0.007 |
| Weekly contact with child | -0.003 | 0.008 |
| Participation in activities | -0.006 | 0.006 |
|  |  |  |
| **CASP-12 Index for Quality of Life** |  |  |
| Depression | -0.042 | -0.012 |
| Experienced loneliness | -0.072 * | -0.025 |
| Having a spouse | -0.011 | -0.011 |
| Having children | 0.055 | 0.025 |
| Weekly contact with child | -0.019 | 0.010 |
| Participation in activities | 0.024 | 0.056 |

Significance * *p* < 0.05, ** *p* < 0.01, *** *p* < 0.00

Data presented are adjusted for demographics and other psychosocial factors.

Full range of the variables were used.
