# Supplementary material for: Do psychosocial factors modify the negative association between disability and life satisfaction in old age?
Source: PLoS One. 2019 Oct 31;14(10):e0224421. doi: 10.1371/journal.pone.0224421 (PMC6822713; doi:10.1371/journal.pone.0224421)
Supplement: S9 Table — Significance * p < 0.05, ** p < 0.01, *** p < 0.00 Data presented are adjusted for demographics and other psychosocial factors. Full range of the variables were used. (DOCX) [file pone.0224421.s009.docx]

**S9 Table. Three-way Interaction Analysis of Disability – Psychosocial Factors – Age on Life Satisfaction and Quality of Life**

|  | **ADL** | **IADL** |
| --- | --- | --- |
| **Life Satisfaction** |  |  |
| Depression | -0.002 | 0.000 |
| Experienced loneliness | -0.000 | 0.000 |
| Having a spouse | -0.009 *** | -0.005 ** |
| Having children | 0.001 | -0.001 |
| Weekly contact with child | 0.008 ** | 0.003 * |
| Participation in activities | -0.001 | -0.001 |
|  |  |  |
| **CASP-12 Index for Quality of Life** |  |  |
| Depression | 0.009 | -0.000 |
| Experienced loneliness | 0.010 | -0.004 |
| Having a spouse | 0.016 * | 0.005 |
| Having children | -0.015 | -0.009 |
| Weekly contact with child | 0.026 *** | 0.008 |
| Participation in activities | -0.015 | -0.010 |

Significance * *p* < 0.05, ** *p* < 0.01, *** *p* < 0.00

Data presented are adjusted for demographics and other psychosocial factors.

Full range of the variables were used.
